# A Unique Presentation of an Undiagnosed Renal Cell Carcinoma

**DOI:** 10.1155/2014/840163

**Published:** 2014-11-06

**Authors:** Georgios Kravvas, Michalis Varnavas, Saad Aldujaily

**Affiliations:** ^1^Department of Urology, Basildon and Thurrock University Hospitals NHS Foundation Trust, Basildon, Essex SS16 5NL, UK; ^2^Urology, Ipswich Hospital, Heath Road, Ipswich, Suffolk IP4 5PD, UK

## Abstract

We describe a 58-year-old lady who presented initially to her general practitioner with a palpable warty urethral nodule. She was subsequently referred to the urology department for further investigations. She underwent flexible cystoscopy and imaging, followed by rigid cystoscopy and excision of the nodule. Histological analysis was consistent with renal cell carcinoma (RCC). CT imaging confirmed the presence of an invading metastatic left renal cell carcinoma with bilateral metastatic deposits to the lungs and adrenal glands. The patient was enlisted on the Panther Trial and received a course of Pazopanib before undergoing radical nephrectomy. Two years later she is still alive with metastases remaining reduced in size and numbers. During this study we have performed a literature review of similar cases with this unusual presentation of RCC.

## 1. Introduction

Renal cell carcinoma metastasising to the ureters and bladder is uncommon but metastasis to the urethra is a rare entity indeed. This case is notable in two ways; firstly, it is only the second available report ever to be published regarding detection of a metastatic urethral nodule from an undiagnosed RCC primary. Secondly, it is the sole of available cases to present directly with a palpable nodule and not indirectly as urinary retention or isolated haematuria.

We describe this very rare presentation of RCC adding to the multitude of symptoms associated with this malignancy. This study also comes to add to the current evidence that urethral metastatic deposits may be best treated with local excision rather than proceeding with more radical interventions. Furthermore we have recorded a positive result of neoadjuvant Pazopanib for metastatic RCC prior to nephrectomy.

## 2. Case Report

A 58-year-old lady with multiple comorbidities presented to her general practitioner with a palpable warty nodule at her urethral meatus. She had a prior 2-year history of malaise and microcytic anaemia. These were attributed to her known history of gastritis, systemic lupus erythematosus, and hypothyroidism.

The urethral nodule had gradually appeared over a course of a few months. There was no history of weight loss or abdominal pain. On examination the abdomen was soft and nontender with no palpable masses. She was thus referred to the urology clinic for further investigations. Five days prior to her appointment she suffered from a single episode of frank haematuria. In view of the haematuria a renal tract ultrasound scan and flexible cystoscopy were organised.

**Figure 1:**
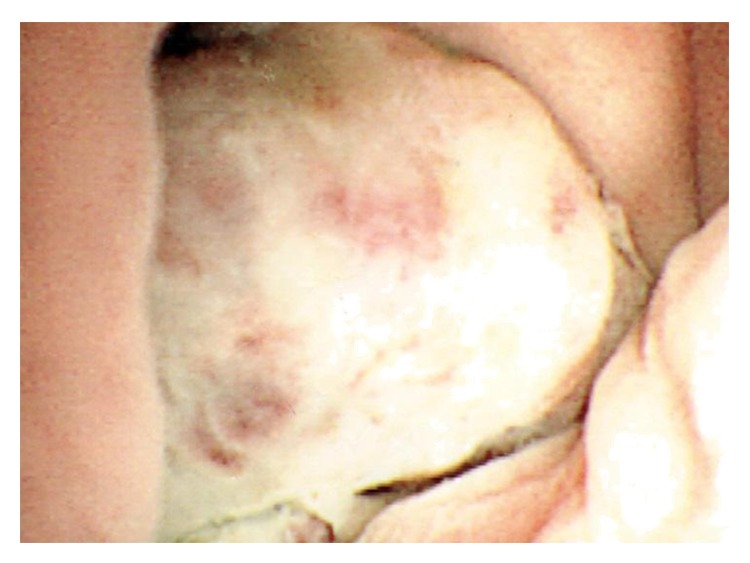
Appearance of the nodule during flexible cystoscopy.

The ultrasound scan of the renal tract demonstrated a single large mass arising from the left kidney, reported at the time as a possible angiolipoma with no other possible findings. A week later flexible cystoscopy confirmed the presence of a warty nodule near the urethral meatus (Figure 1); the rest of the urethra and bladder mucosa were unremarkable.

In view of the findings the patient was put on the waiting list for a rigid cystoscopy and excision of the nodule. In the interim period a CT urogram (Figure 3) was requested and performed revealing a 9 cm mass arising from the upper pole of the left kidney and invading the retroperitoneum. There was also evidence of mediastinal lymphadenopathy along with multiple bilateral adrenal and pulmonary metastases.

**Figure 2:**
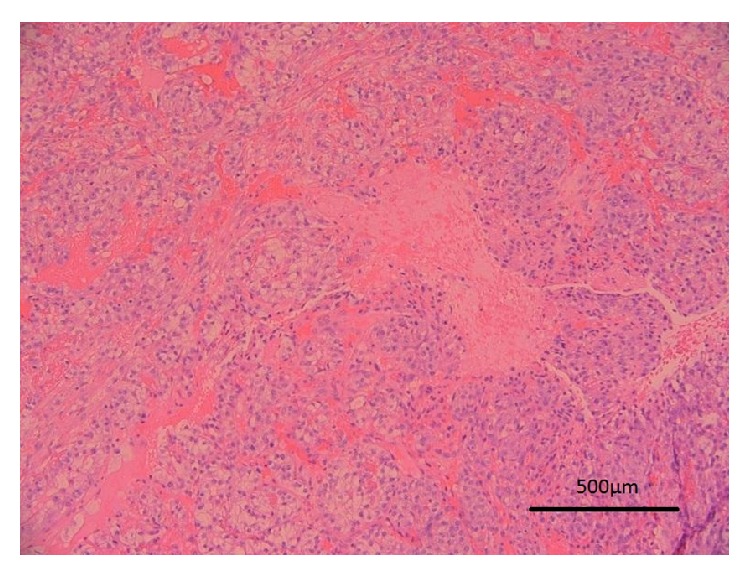
This haematoxylin and eosin stained section shows groups of cells with clear cytoplasmwith a rich associated capillary network consistent with clear cell renal carcinoma.

The patient underwent excision of the nodule the following week. Histological examination of the nodule confirmed the presence of a Fuhrman Grade 3 invading clear cell renal carcinoma (Figure 2).

The results of the CT scan and of the histological analysis were discussed in the urology MDT. It was decided that the patient should be started on the Panther Trial and treated with neoadjuvant Pazopanib, with a view of reducing the tumour size prior to considering surgical resection.

**Figure 3:**
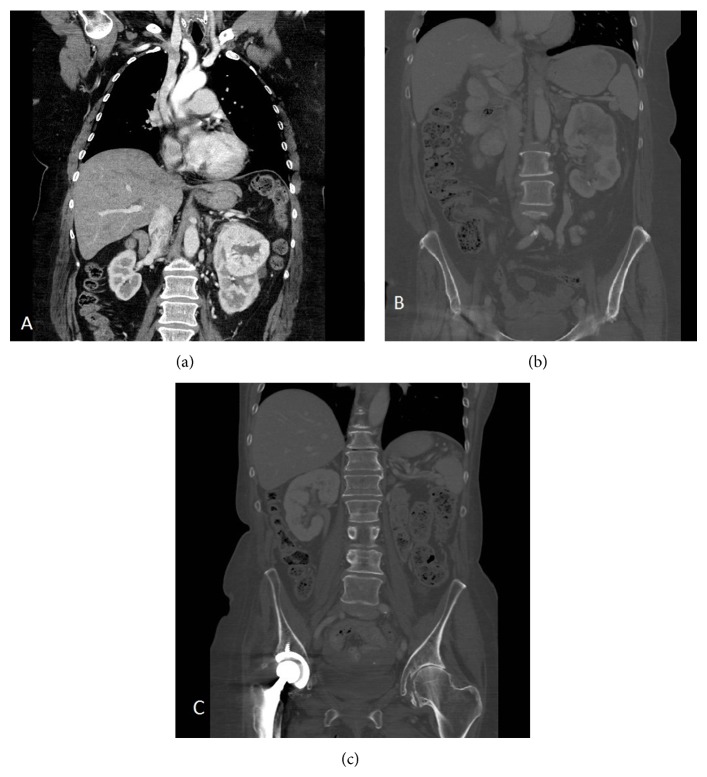
(a) Enhanced CT finding on diagnosis demonstrating the large tumour arising from the upper pole of the left kidney. (b) CT abdomen and pelvis after TKI administration: note the shrinking of the primary tumour. (c) Latest CT abdomen, as part of postoperative follow-up.

A follow-up CT scan (Figure 3) four months later displayed shrinking of the tumour and a decision was then made to proceed with radical nephrectomy. This was performed less than a month later with no intra- or postoperative complications. She was soon restarted on the Pazopanib therapy under the care of the oncology team.

The patient is now two and a half years after the initial diagnosis; she has been followed closely with regular CT scans and flexible cystoscopies. There has been no evidence of local tumour recurrence. Based on imaging, some of the adrenal and pulmonary metastases have disappeared whilst the persisting ones have reduced in size and have remained stable for over a year. There is no current evidence of any new lymph node involvement.

## 3. Discussion

Renal cell carcinomas (RCCs) are a relatively common neoplasm, representing 3% of all adult malignancies. Contrary to popular belief, the triad of haematuria, flank pain, and palpable abdominal mass rarely occur simultaneously. In the majority of cases only one of these symptoms occurs, while being not uncommon to present with a manifestation of metastases or of a paraneoplastic syndrome.

RCC is well known for its tendency to metastasise to other organs. The commonest recipients for metastases are the lungs, liver, bone, and brain. Metastatic deposits to the urethra from a RCC are a remarkably rare occurrence with only seven cases reported in the international literature [1–7]. The route of metastatic spread to the urethra is still unclear, but may include spread via the arterial, venous and lymphatic systems or even by seeding via the urinary tract [1].

Almost all the reported cases (except one) involve late metachronous metastases in patients with a known diagnosis that had already received treatment. This is indeed the first ever documented case to present with a palpable urethral nodule along with visible haematuria, with the only other analogous case having manifested as isolated acute urinary retention before the metastatic deposit being identified on cystoscopy [7].

Prior published cases have reported poor outcomes following the detection of the urethral metastasis, despite transurethral resection or more radical therapeutic interventions such as phalangectomy [5]. In our case the patient was enlisted on the Panther Trial prior to radical nephrectomy; she remains alive and well 2.5 years after diagnosis. Several of the metastases have disappeared. The rest have reduced in size and remained stable for over one year. Local excision of the urethral nodule under rigid cystoscopy was sufficient with no local recurrence having been detected since then. This comes to reinforce the view by Bailie et al. 2013 that radical resection of urethral metastases from RCC is unnecessary and carries significant morbidity with no definite proven benefits.

## 4. Conclusion

- Urethral outgrowths should always undergo excisional biopsy, and although rare, a possibility of a metastatic deposit needs to be considered.
- Metastatic urethral deposits from a RCC primary may be best treated with local excision with regular cystoscopic surveillance rather than with a more radical resection.
